# Efficient Solar Energy Conversion Using CaCu_3_Ti_4_O_12_ Photoanode for Photocatalysis and Photoelectrocatalysis

**DOI:** 10.1038/srep18557

**Published:** 2016-01-04

**Authors:** H. S. Kushwaha, Niyaz A Madhar, B. Ilahi, P. Thomas, Aditi Halder, Rahul Vaish

**Affiliations:** 1 School of Engineering, Indian Institute of Technology Mandi, Himachal Pradesh, India; 2 King Saud University, Department of Physics and Astronomy, College of Science, Riyadh 11451, Kingdom of Saudi Arabia; 3 Dielectric Materials Division, Central Power Research Institute, Bangalore, India; 4 School of Basic Sciences, Indian Institute of Technology Mandi, Himachal Pradesh, India

## Abstract

A highly efficient third generation catalyst, CaCu_3_Ti_4_O_12_ (CCTO) shows excellent photoelectrochemical (PEC) and photocatalytic ability. As only 4% part of the solar spectrum covers UV light, thus it is highly desirable to develop visible light active photocatalyst materials like CCTO for effective solar energy conversion. A direct band transition with a narrow band gap (1.5 eV) was observed. Under light irradiation, high photocurrent density was found to be 0.96 mA/cm^2^, indicating the visible light induced photocatalytic ability of CCTO. Visible light mediated photocatalytic and photoelectrocatalytic degradation efficiency of CaCu_3_Ti_4_O_12_ pellets (CCTO) was investigated for three classes of pharmaceutical waste: erythrosin (dye), ciprofloxacin (antibiotic) and estriol (steroid). It is found that the degradation process follows first order kinetic reaction in electrocatalysis, photocatalysis and photoelectrocatalysis and high kinetic rate constant was observed in photoelectrocatalysis. This was quite high in comparison to previously reported methods.

Photocatalysis has attracted great interest in recent years due to its potential to degrade various pollutants into environmental-benign chemicals at low cost^1^,^2^,^3^. Photoelectrochemical (PEC) water splitting along with photocatalytic oxidation is a sustainable process for the conversion of solar energy for energy production. First time in 1972, Fujishima and Honda demonstrated the prospect of creating a clean and sustainable fuel through PEC process^4^. The other aspect of using this solar energy is the degradation of harmful, widely used pharmaceutical wastes in to non- toxic chemicals in the process of water treatment. Pollutants from pharmaceuticals and industrial wastages have serious and long-term effects on human health and aquatic environment. These pollutants enter into the environment through the discharge of manufacturing plants, industrial waste water and sewage treatment plants (STP). Pharmaceutical waste water contains dyes, antibiotics, estrogens, steroids and other drugs^5^,^6^,^7^. Fluoroquinolone antibiotics and estrogenic hormones such as ciprofloxacin, norfloxacin, enrofloxacin, 17β- estradiol (E2),17α- ethylestradiol, and estriol are largely used to treat diseases and infection. However, incomplete degradation of these drugs releases harmful residual components in surface water^8^,^9^. Similarly, a low concentration of antibiotics, which are commonly used in agriculture to promote growth and prevent disease, can cause proliferation of drug resistant bacteria causing a potential threat for human health and ecosystem^10^,^11^. For example, Erythrosine which is extensively being used for artificial coloring for foods and drugs like ibuprofen, its toxicological test shows sensitivity to light and increase thyroid hormone levels and lead to hyperthyroidism^12^,^13^. Thus alternative cost-effective techniques are highly required to degrade this environmental discharge of pharmaceuticals and hazardous dyes.

A PEC cell provides a sustainable process to transform solar energy into chemical fuel and electricity. This is a highly efficient operation can be triggered simply by sunlight. PEC water splitting is the most direct mechanism which can be used for hydrogen generation, solar energy harvesting and waste water treatment. Water splitting is an endergonic process requiring an energy input of 237 kJ/mol of water. Solar light can be an efficient source for the necessary energy input. However, that energy needs to be efficiently collected through absorption processes and transferred into a water molecule to break its chemical bonds. Photoelectrochemical splitting of water into oxygen and hydrogen has emerged as a potentially viable option for the efficient degradation of pollutants.

Many of the first generation photocatalyst like metal oxides have been used for photoelectrochemical solar cell and photocatalytic degradation of antibiotics, endocrine disrupting chemicals (EDCs) and various pollutants but they have limitations like low yield and redistricted catalysis process occuring only in UV irradiation^14^,^15^,^16^. Various strategies have been used to develop visible light active photocatalysts, e.g. metal (Cr, Co, V and Fe) doping, non-metal (N, F, S, and C) doping, dye sensitization and doping of supported nanoparticles^17^,^18^,^19^,^20^,^21^. These systems are classified as second generation of photocatalyst materials. Most of these second generation of photocatalysts have the inefficiency due to instability and charge recombination processes. Thus large interest arose for the third generation photocatalysts which have multiphoton excitation due to heterojunction of low band gap photoactive materials and TiO_2_ 22,23. ABO_3_ perovskite and layered structures have great potential for the visible light photocatalysis and considered as third generation photocatalyst. The band structure of these materials can be tailored by substitution of A and B sites^24^. The double perovskite structures like A_2_InNbO_6_ and A_3_CoNb_2_O_9_ have shown their potential for water splitting under visible light^25^,^26^,^27^.

The photocatalytic and photoelectrochemical performance of a catalyst mainly depend upon (i) the light absorption properties, (ii) reduction and oxidation rates on the surface by the electron and hole, and (iii) the electron-hole recombination rate. CaCu_3_Ti_4_O_12_ (CCTO) is a combination of well-known highly UV active photocatalyst TiO_2_ and visible light absorbing CuO. CaCu_3_Ti_4_O_12_ (CCTO) is a cubic double-perovskite which has Cu^+2^ and Ca^+2^ on A site and Ti^+4^ on the B site. It is extensively studied for the energy storage and microelectronics applications due to its high dielectric constant^28^,^29^,^30^,^31^. In this paper, we demonstrate the visible light induced photoelectrochemical behavior and photocatalytic activity of CCTO ceramic for the three different classes of pharmaceuticals: dyes, antibiotics and steroid hormones. To the best of our knowledge, this is the first report for the enhanced visible light photocatalytic activity of CCTO ceramic pellets for the pharmaceutical pollutants.

## Results and Discussion

**Figure 1:**
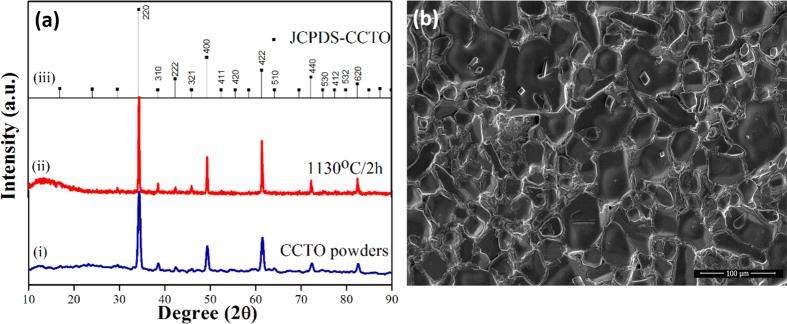
(**a**). X-ray diffraction patterns of (i) as prepared CaCu_3_Ti_4_O_12_ (CCTO), (ii) CCTO pellets sintered at 1130 ^o^C/2 h and (iii) ICDD data file card no. 01-075-1149 for CCTO. (**b**) Scanning electron micrographs of the CCTO pellets sintered at 1130 °C/2 h.

Ceramic samples of CCTO prepared from the oxalate precursor route had been studied by different characterization techniques. The X-ray diffraction patterns were recorded on the as prepared CCTO powders (Fig. 1a(i)), the pellets sintered at 1130 °C (Fig. 1a(ii)) are compared well with ICDD data (01-075-1149) (Fig. 1a(iii)), demonstrating the single phase nature of CCTO. In the XRD- pattern of sintered CCTO pellet, there is not any peaks for CuO and TiO_2_ which shows that phase pure CCTO can be synthesized using this process. The crystallite size was calculated using Scherer formula and observed as 26 nm for powder and 64 nm for sintered pellets of CCTO. The heat treatment around 1130°C resulted sintering of the particles and increment in crystallite size. Figure 1b shows the SEM micrograph of CCTO pellets sintered at 1130 °C for 2 h.

**Figure 2:**
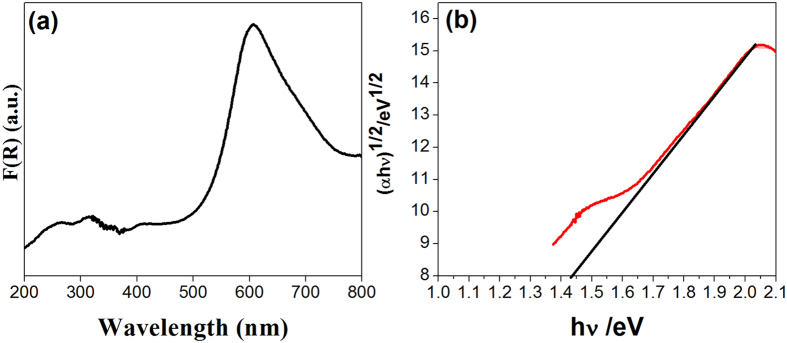
(**a**) UV-Vis Absorbance spectrum of the CCTO (**b**) plot between *Kubelka-munk* function (*F*(*R*) *hʋ*)^*2*^ and photon energy (*hʋ*) shows direct allowed transition.

The optical properties of CCTO had been studied using UV-visible spectroscopy^32^ and Fig. 2a shows the diffuse reflectance spectra for the CCTO powder. A broad absorption band was observed between 200–700 nm with highest absorption at 305 nm. The absorption cutoff wavelength for the CCTO powder is 750 nm. Figure 2b shows the plot between Kubelka-Munk function (*F*(*R*)*hʋ*)^1/ 2^ and photon energy (*hʋ*)^33^. A direct transition of 1.5 eV was estimated from the tangent line in the plot which corresponds to the visible light absorption and low band gap of CCTO powder. In CCTO, three possible mechanisms are attributed for photogenerated hole formation for 3d^9^ (i) d_x2-y2_ → d_xy_ (ii) d_x2-y2_ → d_z2_ (iii) d_x2-y2_ → d_zx, yz_. *Clark et al.* reports the visible light activity of Pt-CCTO complex for dye degradation^32^. The observed absorbance data shows that CCTO has a direct transition in the visible region. In CCTO, the higher energy absorption edge arises due to direct transition from Cu *3d*- O *2p* hybridized valance band to the Ti-*3d* conduction band and the lower energy absorption is attributed as transition between valance band and unoccupied Cu *3d* band. CCTO have visible light activity due to photo-induced charge transition from Cu^2+^, Ti^4+^ ground state to Cu^3+^, Ti^3+^ excited state^32^. The visible light activity and the photo induced charge transfer show its potential for photoelectrochemical solar cell and photocatalytic pollutant removal process. CCTO has large absorption spectrum in visible region which is favorable for activity of CCTO as catalyst under visible light. Therefore, CCTO was used for pollutant degradation and photoelectrodes under visible light irradiation.

**Figure 3:**
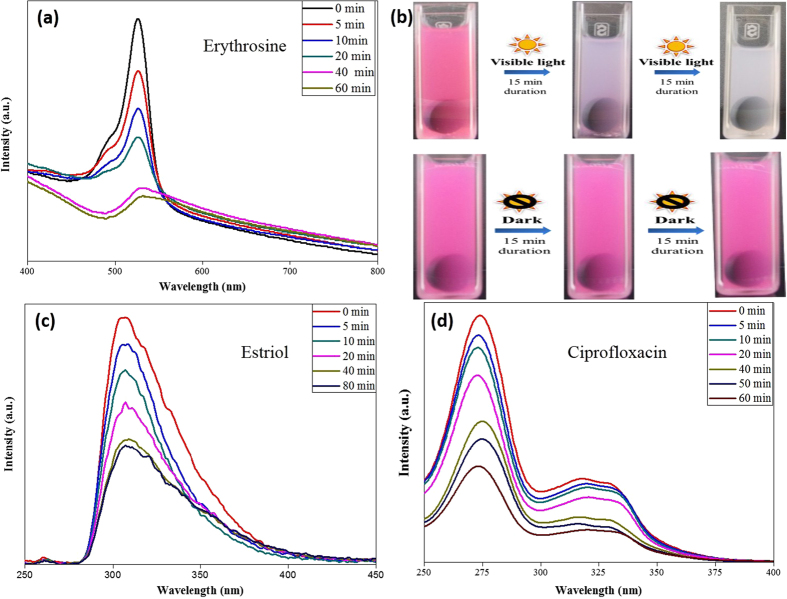
(**a**) Change in absorbance of Erythrosine (525 nm) (**b**) Color change in erythrosine under dark and light (Figure drawn by author H.S. Kushwaha) (**c**) Change in absorbance of Ciprofloxacin (276 nm) and (d) fluorescence emission of estriol (305 nm excited at 240 nm) with time in photocatalytic degradation using CCTO pellets under visible light (λ>420 nm).

The photocatalytic activity of as prepared CCTO pellet was evaluated by the degradation of erythrosine, ciprofloxacin and estriol. Fig. 3a shows the variation in absorbance spectra of an aqueous solution of erythrosine in the presence of CCTO pellets irradiated by a visible light source. The absorption peak corresponding to erythrosine dye was observed at 525 nm and the peak intensity decreases rapidly with the prolonged exposure time. The pink solution of erythrosine turned into colorless solution after only 40 minute exposure. Erythrosine dye molecules react with ˙O_2_ or ˙OH radicals generated during splitting of H_2_O in photocatalysis process which further perform mineralization of erythrosine molecules^34^. Figure 3b shows the images of time dependent degradation of erythrosine in the presence of CCTO. Under visible light irradiation, the pink color of the erythrosine started fading within 15 minutes and turned colorless within 30 minutes. However, in the dark, we did not observe any detectable color change. That indirectly proves that CCTO is photocatalytically degrading the erythrosine molecules. Figure 3c shows the change in concentration of ciprofloxacin aqueous solution during photocatalysis with CCTO pellet under exposure of visible light. The results show that the absorption peak of ciprofloxacin molecules decreases with the irradiation time at 276 nm. Photocatalytic degradation of estriol using CCTO pellet was analyzed by fluorescence spectroscopy. Estriol shows a strong emission peak at 306 nm when excited with 240 nm source wavelength. Figure 3d shows the rapid decrease in fluorescence intensity of estriol molecules during photocatalysis using CCTO pellets under visible light exposure which attributed as degradation in concentration of estriol molecules.

**Figure 4:**
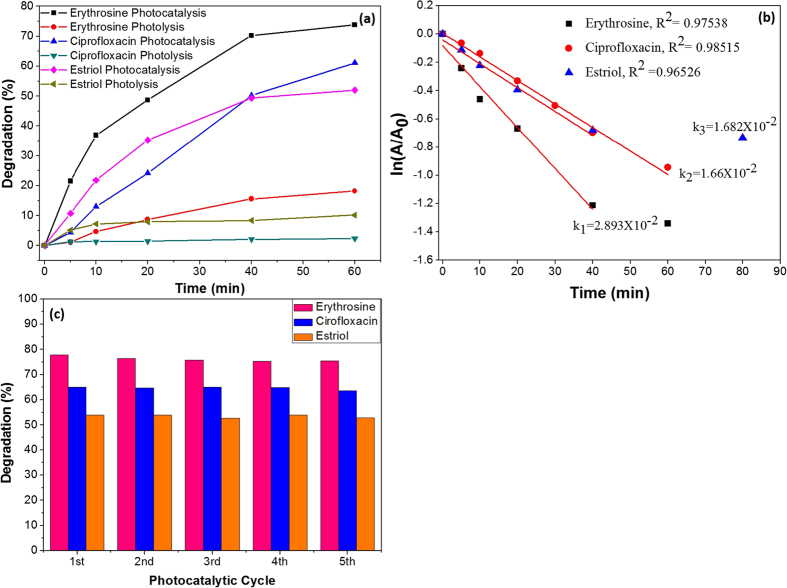
(**a**) Degradation rate (%), (**b**) first order kinetic decay plots for the visible light catalysis of erythrosine, ciprofloxacin and estriol, (**c**) Degradation rate (%) in different photocatalytic cycle.

The degradation efficiency (D %) of CCTO pellets for the pollutants is calculated using


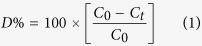


where C_0_ is the initial concentration of drug and *C*_*t*_ the concentration of drug after time (*t*) exposure. Figure 4a shows the comparative degradation (%) of erythrosine, ciprofloxacin and estriol in photolysis and photocatalysis under the visible light exposure (λ>420 nm). In the photolysis, no significant degradation was observed for all the pollutants. CCTO photocatalytic pellet shows maximum degradation efficiency for the erythrosine dye under visible light which is better than previous reported methods^34^,^35^. The dye molecules degraded up to 70% in visible light irradiation of 40 min while under dark light no degradation of pollutant was observed. The prepared CCTO ceramic pellets degrade 60% of ciprofloxacin concentration in 60 min while for estriol molecules, CCTO pellet saturates earlier. Upto 50% degradation was performed in 40 min using prepared CCTO pellets which are higher than earlier used catalysts for visible light mediated degradation^36^,^37^,^38^.

The kinetic behavior of CCTO photocatalyst for the degradation of erythrosine, ciprofloxacin and estriol were investigated using Langmuir-Hinshelwood kinetic equation^39^:


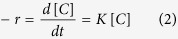



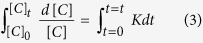



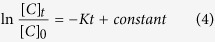


where *C*_*0*_ and *C*_*t*_ are the initial concentration and concentration after time t for pollutants. *K* is the first order catalytic rate constant which is represented by slope of the ln (*A*_*t*_*/A*_*0*_) and degradation time (*t*) plot. Figure 4b shows the kinetics decay plots for the degradation of erythrosine, ciprofloxacin and estriol. All three of the pollutants show first order decay for the visible light catalysis using CCTO ceramic pellets. The kinetic rate constants for the erythrosine, ciprofloxacin and estriol are calculated as *k*_*1*_ (0.0289 min^−1^), *k*_*2*_ (0.01682 min^−1^) and *k*_*3*_ (0.0166 min^−1^). Figure 4c shows the degradation rate for all the three pollutants in the repeated photocatalytic process using CCTO pellet as catalyst. In all the five cycle, constant degradation rate was observed which attributed as stability and reusability of the catalyst.

**Figure 5:**
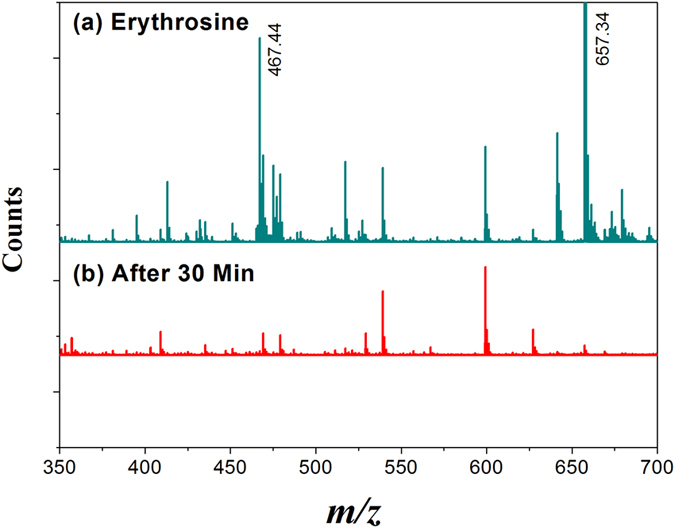
Mass spectra of (**a**) the erythrosine solution before photocatalysis, (**b**) after 30 minutes photocatalysis with CCTO pellet under visible light.

Mass spectroscopy was used to probe the generated byproduct after photocatalysis of erythrosine. Figure 5 shows the mass spectra for erythrosine aqueous solution before and after photocatalysis. The intermediates with *m/z* 467.44 and 657.34 corresponding to the fragments of erythrosine and gradually disappeared after irradiation for 30 minutes in presence of CCTO. Photocatalytic degradation pathway of erythrosine can be analyzed from the transformation of the intermediate species analyzed in mass spectroscopy^40^. Erythrosine dye molecules reacts with photogenerated holes and 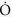
_2_ or ˙OH radicals which are generated during water splitting. Erythrosine transformed into 2,5-dihydroxybenzoic acid and phthalic acid^41^,^42^. The intermediate products further react with ∙OH radicals and converted into CO_2_ and water by ring opening and mineralization process^43^.

In the photocatalytic degradation of ciprofloxacin both ˙OH radical and photogenerated holes play role in complete oxidation of molecule and intermediates. The photogenerated holes attack on the N12 position in ciprofloxacin. On the other hand, the addition of ˙OH radical occurs at C10 and C5 position in quinolone ring. The intermediates formed in the process are further oxidized with hydroxyl radicals and perform a complete oxidation in CO_2_^44^. The conduction band in CCTO which contributed by Ti (*3d*) state, plays important role to return the quinolone molecule in ground state by transferring electron into conduction band after photo-excitation. The electron in conduction band further transferred to donor quinolone molecules^45^. In the photocatalytic degradation process of estriol, the active ∙OH radicals formed at photocatalyst surface by water splitting. These ∙OH radicals are more likely to attack the benzene ring at C2 and C4 of estriol followed by reaction with oxygen and elimination of HOO∙ group^46^,^47^. 10ε-17β-dihydroxy-1, 4- estradien-3-one (DEO) and testosterone like species are generated as intermediate products in photocatalytic degradation of estriol. These species are further completely mineralized into CO_2_. The intermediates and final products did not have any estrogenic activity due to the complete dissociation of phenol group which is highly responsible to initiate estrogenic activity^48^.

**Figure 6:**
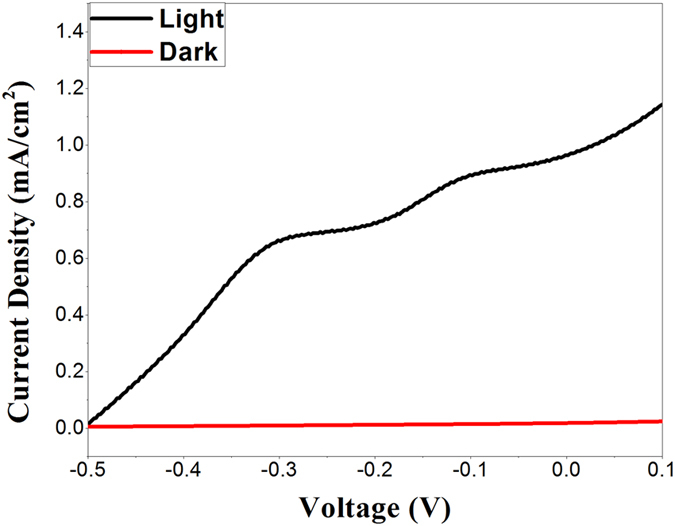
(**a**) PEC studies of CCTO in 1 M L^−1^ KOH under visible light illumination and in the dark.

To demonstrate the enhanced electron transfer in the CCTO system, some PEC tests were carried out. The photoelectrochemical activity of CCTO under visible light was analyzed by measuring the photocurrent using linear sweep voltammetry (LSV) in the dark and under visible light irradiation as shown in Fig. 6. Cyclic voltammetry observations attributed as high stability of CCTO photo-anode in the electrolyte during electrochemical measurements. The higher photocurrent density was observed under the light illumination for the CCTO pellets which was attributed as light harvesting ability and narrow band gap of CCTO. The photocurrent density (*J*_*sc*_) was observed as 0.97 mA/cm^2^ under light irradiation. The interband electron transition due to photon absorption during visible light irradiation results into the generation of photocurrent and four fold increase of current density. LSV and cyclic voltammetry showed that the CCTO can be used effectively as photocatalysts and photoelectrode materials.

In order to understand the origin of photoactivity enhancement in the PEC system, the flat band potential was measured. Here, the flat band potential of electrodes was determined by the Mott–Schottky relation^1^:


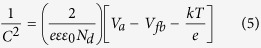


Where C = space charge layers capacitance, e = electron charge, ε = dielectric constant, ε_0_ = permittivity of vacuum, N_d_ = electron donor density,V_a_ = applied potential and V_fb_ =  flat band potential.

The flat band potential (V_fb_) was determined by taking the x intercept of a linear fit to the Mott–Schottky plot (See supporting information, Fig. S1), 1/C^2^, as a function of applied potential (V_a_). The apparent donor density of the CCTO photoanode was calculated as 1.9×10^15^. Flat band potential of 0.4 V *vs* SCE was observed for CCTO photoanode. Electrochemical impedance spectroscopy (EIS) was performed to investigate the electrochemical behavior of CCTO photoanode. Figure S2 (See supporting information) shows EIS results presented in the form of Nyquist plot. CCTO electrode exhibits R_ct_ (2.8 kΩ) indicating the more favorable environment for hole transfer to the electrolyte.

**Figure 7:**
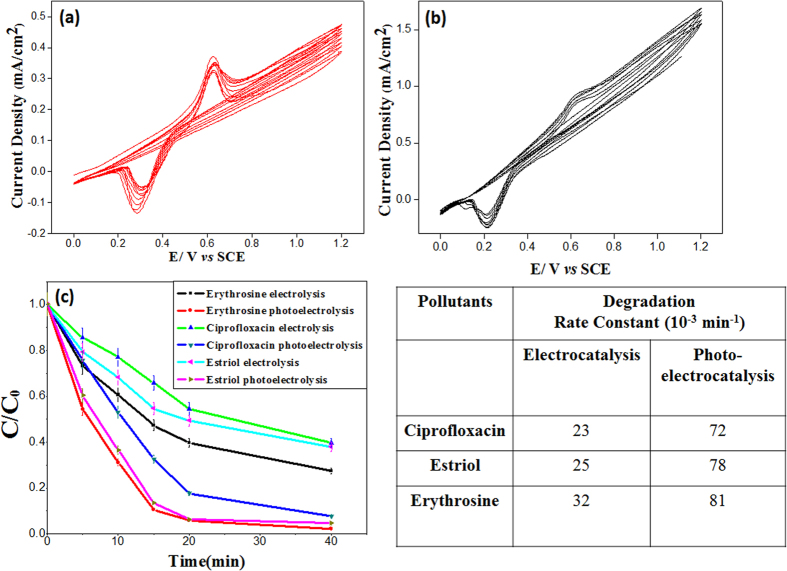
Cyclic voltammogram for (**a**) electrocatalysis (**b**) photoelectrocatalysis of erythrosine solution (The arrows in figure show the change in the peak current for the oxidation for pollutants). and (**c**) First order kinetic decay plots for the electrocatalysis and photoelectrocatalysis of erythrosine, ciprofloxacin and estriol.

Figure 7a,b show the electrocatalytic and photoelectrocatalytic degradation of erythrosine by CCTO. The cyclic voltammogram data (Fig. 7a,b) show a sharp oxidation peak at 0.62 V (vs SCE). This peak intensity decreased on the progression of scans (with the increased number of cycle). The decrease in the anodic peak current can be attributed as oxidative degradation of erythrosine on CCTO electrode as the visible change in the color of the electrolyte was also observed. Careful investigations of the CCTO pellet after electrochemical and photoelectrochemical studies shows no noticeable change(see supporting information), indicating that decline of the peak intensity in CV was due to the degradation of electrolyte materials and not accountable for the structural or chemical degradation of CCTO. The photoelectrochemical performance of CCTO pellets shows significantly higher current density during the visible light assisted electrocatalytic degradation (Fig. 7b). In photoelectrochemical study, higher degradation rate was observed in comparison to the electrochemical degradation of erythrosine. Figure 7c shows the degradation plots for electrocatalysis and photoelectrocatalysis of erythrosine, ciprofloxacin and estriol. The kinetic decay constants for the degradation of all three pollutants was found to be higher under photoelectrocatalytic degradation which is attributed as role of photocurrent in generation of more OH˙ radicals for catalysis and effective separation of charge carriers. The stability of the catalyst has been verified using XRD before and after catalysis. The XRD data shows indifference with the pre-catalysis data collected as shown in Fig S3. (See supporting information).

The photocatalytic activity is controlled by both the ability of light-harvesting and the separation of e^−^/h^+^ pair. The e^−^/h^+^ pair generated due to light absorption will recombine if they are not separated quickly. A high photocurrent indicates that CCTO has a strong ability to generate and transfer the photo-excited charge carriers and rapidly production of ∙OH radicals during electrochemical water splitting under light illumination. These ∙OH radicals plays major role in the photocatalytic degradation of pollutants. Time-dependent fluorescence experiments had been carried out to identify these ∙OH (See supporting information, Fig. S4). Previous reports and the experimental results indicated that photocatalytic degradation of pharmaceuticals initiated by excitation of electron form the valance band to the conduction band after irradiation of light^2^,^14^,^49^. The photocatalytic performance of catalyst is depends upon its light absorption ability and electron transfer ability. The process starts only when the incident light energy is equal to or larger than band gap. CCTO photocatalyst is a novel material for visible light catalysis due to its high absorbance spectrum, narrow band gap and ability to transfer the photogenerated charge carriers. The process followed by the direct oxidation of pharmaceuticals by photo generated hydroxyl radicals and holes. The indirect oxidation is performed by the ˙OH radials generated by water splitting.

## Conclusion

In conclusion, a novel visible light active CaCu_3_Ti_4_O_12_ ceramic was synthesized from the oxalate precursor route. CCTO have broad absorbance spectrum for visible light and narrow band gap which makes it a potential material for visible light induced photocatalysis and photoelectrochemical cell. Under visible light irradiation, CCTO pellet electrodes show high photocurrent density 0.97 mA/cm^2^. It shows the potential of CCTO photoanode for the high performance PEC solar cells for energy conversion. CCTO ceramic pellets have more efficient photocatalytic ability to degrade erythrosine, ciprofloxacin and estriol than any other catalyst under visible light. The catalysis was performed by using CCTO pellets and does not require any additional filtration process to remove the catalyst which is an added advantage over the catalysis in powder form. This study has demonstrated fabrication of third generation photocatalyst with two distinct transition metals for the visible light mediated photoelectrochemical and photocatalytic degradation for three different classes of pharmaceutical water pollutants which can be applied to develop new class of visible light active catalysts for other pollutants.

## Methods

### Synthesis of CaCu_3_Ti_4_O_12_

Ceramic samples of CaCu_3_Ti_4_O_12_ (CCTO) were prepared from the oxalate precursor route^28^. In a typical preparation, initially, the titania gel was prepared from the aqueous TiOCl_2_ (0.05 M) by adding NH_4_OH at room temperature till the pH reaches ~8.0 and washed with water to remove NH_4_Cl. Synthesized titania gel was powdered and dissolved in H_2_C_2_O_4_.2H_2_O. In the obtained solution, calcium carbonate was added and stirred. The solution remained clear without any precipitate formation. Cupric chloride solution in acetone and water (80:20) was added slowly and stirred continuously at 10 °C. The thick precipitate was separated, washed several times with acetone to make it chloride-free and dried in air. The precipitate thus prepared was isothermally heated above 680 °C to get the ceramic powders of CaCu_3_Ti_4_O_12._ The resultant powder was ground thoroughly, ball milled for 2 h and granulated by adding polyvinyl alcohol (PVA) and poly ethylene glycol (PEG), then pressed into pellets at 150 MPa with a diameter of 12 mm and thickness of 2 mm. The green pressed pellets were slowly heated to 600 °C to get rid of the binder. Finally, the pellets were sintered in air at 1130 °C for 2 h. Pellet densities were measured by the Archimedes principle using xylene as the liquid medium.

### Material Characterization

After sintering, X-ray powder diffraction studies were carried with an X’pert diffractometer (Philips, Netherlands) using Cu Kα_1_ radiation (λ = 0.154056 nm) in a wide range of 2θ (10^o^–90^o^) with 0.02 step size to examine the phase constitutes of the specimens. Scanning electron microscope (FEI-Technai SEM-Sirion) was used to observe the microstructure of the sintered pellets. Optical properties of CCTO powder were analyzed by UV-vis absorbance spectroscopy.

### Photoelectrochemical Characterization

A scanning potentiostat (Metrohom, Autolab) was used to perform cyclic voltammetry and electrochemical impedance studies in a three-electrode photoelectrochemical cell with CCTO pellet as the working electrode, a calomel electrode as the reference, a platinum wire as the counter electrode and 1M L^−1^ KOH as electrolyte. To fabricate the working electrode, a copper wire was attached with the silver paste at side of pellet and sealed with epoxy to avoid the copper contact with electrolyte. Cyclic voltammetry and Linear Sweep Voltammetry (LSV) scan were performed between −1 V to 0.5 V *vs.* SCE at scan rate of 2 mV s^−1^. For photocurrent measurements, PEC cell was assembled using a CCTO pellet electrode with an area of 1 cm^2^ as a photoanode, Pt wire as a counter electrode and 1 M L^−1^ KOH as electrolyte. A halogen lamp with a power of 100 mW/cm^2^ was used as the incident light source and placed at 10 cm distance from the electrochemical cell.

### Visible Light Photocatalysis

Pharmaceutical dye erythrosine, antibiotic ciprofloxacin and estriol were used to test the photocatalytic activity of sintered CCTO pellets. Table 1 shows the chemical structures, applications and toxicological effect of pollutants which are used for photocatalytic degradation in this paper The photocatalytic degradation experiments was carried out by using 10 ml solution of10 mg/L ciprofloxacin, 10 mg/L erythrosine and 1 mg/L of estriol in deionized water. A total of 10 ml solution of pharmaceutical compound was transferred in pyrex glass vessel and the sintered CCTO pellet was placed vertically in vessel. The vessel was kept in dark for 30 min to allow stabilization and pre adsorption of pharmaceutical species on catalyst pellet. The photocatalytic degradation process was initiated by exposing the reaction vessel with visible light 150 W halogen lamp with UV-light cutoff filter (λ>420 nm). During light exposure, cold water was circulated around the vessel to maintain at room temperature. Sample aliquots of pharmaceuticals were collected at various time intervals and analyzed for change in concentration. The concentration of erythrosine and ciprofloxacin were analyzed using Shimadzu-2450 UV-vis spectrophotometer by measuring the peak intensity. The degradation of estriol was investigated by fluorescence spectrophotometer. The analysis was performed using 3 ml of sample aliquots. High resolution mass spectroscopy analysis was performed to analyze the subsequent species produced during photocatalytic degradation of erythrosine.

### Photoelectrocatalysis

Photoelectrocatalytic degradation of erythrosine was carried out in a single photoelectrochemical compartment. The CCTO photoanode (12 mm diameter and 1 mm thickness) and a platinum wire cathode were placed in parallel in a cuboid glass reactor (volume of 100 mL) with a SCE reference electrode. All electrodes were connected to a Metrohom, Autolab electrochemical station. A halogen lamp placed outside the glass reactor and paralleled to the CCTO photoanode. A bias potential applied on the CCTO photoanode was varied from 0.5 V to 1.5 V (vs SCE) under visible light intensity of 100 mW cm^−2^. All the experiments were performed at room temperature (about 25 °C) with a magnetic stirrer at a constant speed.

## Additional Information

**How to cite this article**: Kushwaha, H. S. *et al.* Efficient Solar Energy Conversion Using CaCu_3_Ti_4_O_12_ Photoanode for Photocatalysis and Photoelectrocatalysis. *Sci. Rep.* **6**, 18557; doi: 10.1038/srep18557 (2016).

## Supplementary Material

Supplementary Information

## Figures and Tables

**Table 1:**
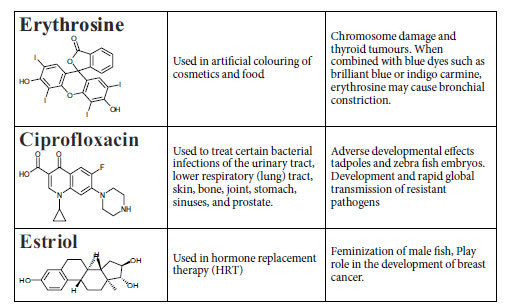
Applications and toxicological effects of chemicals under study.
