# Supplementary material for: Efficient Solar Energy Conversion Using CaCu3Ti4O12 Photoanode for Photocatalysis and Photoelectrocatalysis
Source: Sci Rep. 2016 Jan 4;6:18557. doi: 10.1038/srep18557 (PMC4698584; doi:10.1038/srep18557)
Supplement: Supplementary Information [file srep18557-s1.doc]

*Supporting Information*

**Efficient Solar Energy Conversion Using CaCu_3_Ti_4_O_12_ Photoanode for Photocatalysis and Photoelectrocatalysis**

H. S. Kushwaha^1^, Niyaz A Madhar^2^, B. Ilahi^2^, P. Thomas^3^, Aditi Halder^4^, Rahul Vaish*^1^

^1^School of Engineering, Indian Institute of Technology Mandi, Himachal Pradesh, India.

^2^King Saud University, Department of Physics and Astronomy, College of Science, Riyadh 11451, Kingdom of Saudi Arabia.

^3^Dielectric Materials Division, Central Power Research Institute, Bangalore, India.

^4^School of Basic Sciences, Indian Institute of Technology Mandi, Himachal Pradesh, India

*Corresponding Author, Email: rahul@iitmandi.ac.in, Phone: +91-1905-237921, Fax: +91-1905-237945

Fig. S1. Results of Mott-Schottky analysis for the CCTO photoanode.

Electrochemical Impedance Spectroscopy:

To further analyze the electrochemical behavior of the solar cells, electrochemical impedance spectroscopy (EIS) measurements were performed under dark and illumination[^2^](#_ENREF_2). We selected the Randles-Ershler model for the fitting of experimental data, in which R_s_ is the electrolyte resistance, CPE and R_ct_ are the capacitance phase element and the charge transfer resistance across the interface of the semiconductor electrode and the electrolyte, respectively. CCTO electrode exhibits R_ct_ (2.8 kΩ) indicating the more favorable environment for hole transfer to the electrolyte.

Fig S2. Nyquist plots of the CCTO photoanode under dark and light illumination and the equivalent circuit model used for fitting the experimental data.

Fig. S3. X-ray diffraction pattern of CCTO pellet befor and after catalysis (photocatalysis + photoelectrocatalysis) process.

Analysis of hydroxyl radical (•OH)

The formation of hydroxyl radicals (•OH) at photo-illuminated CCTO interface was detected by the PL technique using terephthalic acid as a fluorescence probe molecule. Terephthalic acid readily reacts with •OH to produce highly fluorescent product, 2-hydroxyterephthalic acid[^3^](#_ENREF_3). This method relies on the PL signal at 425 nm of the hydroxylation of terephthalic acid with •OH generated at the catalyst/water interface. The amount of •OH radicals produced during photocatalysis is proportional to the photoluminescence intensity of 2-hydroxyterephtalic acid. This experimental procedure is similar to the measurement of photocatalytic activity except replacing the aqueous solution by the 5 × 10^−4^ M terephthalic acid aqueous solution with a concentration of 2 × 10^−3^ M NaOH. The PL spectra of generated 2-hydroxyterephthalic acid were measured on an Agilent fluorescence spectrophotometer. After visible-light irradiation for every 20 min, the reaction solution was used to measure the increase in the PL intensity at 425 nm excited by 315 nm light.

Fig S4 shows the changes in the PL spectra of terephthalic acid solution under visible light with increasing irradiation time. A gradual increase in the PL intensity at about 425 nm is observed. However, No change is observed in PL intensity in the absence of visible light irradiation. This indicates that photoluminescence intensity increases due to luminescent 2-hydroxyterephthalic acid (TAOH) produced by chemical reaction between TA and •OH formed during photocatalytic process.


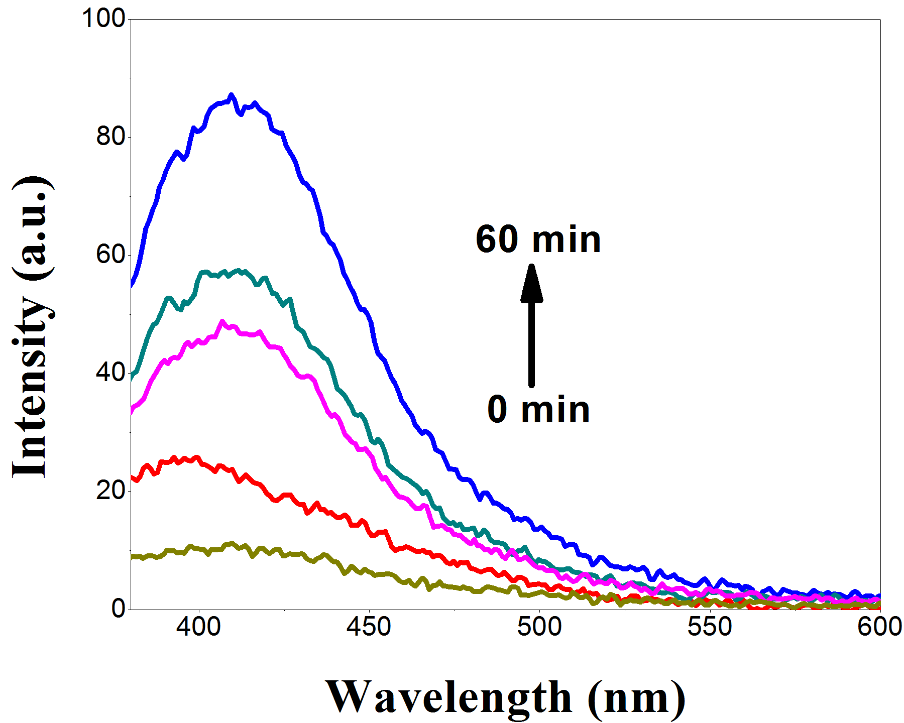


Fig. S4. PL spectral changes with visible light irradiation time for CCTO in a 5 × 10^−4^ M basic solution of terephthalic acid.

1. Cardon, F. & Gomes, W. On the determination of the flat-band potential of a semiconductor in contact with a metal or an electrolyte from the Mott-Schottky plot. *J. Phys. D* **11**, L63 (1978).

2. Wang, Q., Moser, J.-E. & Grätzel, M. Electrochemical impedance spectroscopic analysis of dye-sensitized solar cells. *J. Phys. Chem B* **109**, 14945-14953 (2005).

3. Yu, J., Wang, W., Cheng, B. & Su, B.-L. Enhancement of photocatalytic activity of mesporous TiO2 powders by hydrothermal surface fluorination treatment. *J. Phys. Chem. C* **113**, 6743-6750 (2009).
